# The global burden of stomach cancer and its risk factors from 1990 to 2021: findings from the Global Burden of Disease Study 2021

**DOI:** 10.1186/s12889-025-23901-y

**Published:** 2025-08-06

**Authors:** Le Zhou, Bo Han, Yinjiao Yuan, Zhuowei Dong, Yijing Shi, Ruinian Zheng

**Affiliations:** 1 Shenzhen School of Clinical Medicine, Southern Medical University, Shenzhen, Guangdong 518000 China; 2 Department of Oncology, Dongguan Key Laboratory of Precision Diagnosis and Treatment for Tumors, Dongguan Institute of Clinical Cancer Research, The Tenth Affiliated Hospital, Southern Medical University (Dongguan People’s Hospital), Dongguan, 523059 China; 3 Phase I Clinical Trial Center, The Tenth Affiliated Hospital, Southern Medical University (Dongguan People’s Hospital), Dongguan, 523059 China

**Keywords:** Stomach cancer, GBD, Risk factors, Incidence, DALYs

## Abstract

Background: Stomach cancer is one of the leading cancers worldwide. This article aims to analyze and assess the global burden of stomach cancer from 1990 to 2021, identify its main risk factors, and make projections for 2030 using the newly published 2021GBD database. Methods: In the Global Burden of Disease Study 2021, we analyzed the incidence, death, and DALYs related to stomach cancer. Age-standardized incidence rate (ASIR), age-standardized death rate (ASDR), and age-standardized DALYs rate were obtained and analyzed. Results: The incidence of stomach cancer increased from 980,899 in 1990 to 1,230,233 in 2021. ASIR has declined from 24.76 to 14.33 per 100,000 people. ASDR dropped from 22.01 to 11.20 per 100,000 people. The global DALYs for stomach cancer was 22,786,633, and the age-standardized DALYs rate was 262.75 per 100,000 people. Men have higher rates of ASDR, ASIR, and age-standardized DALYs compared to women. In 2021, East Asia have the highest burden of stomach cancer associated with behavioral, high-sodium diets, smoking, and dietary habits. Projections for 2030 indicate that ASIR, ASDR, and age-standardized DALYs rates will all continue to decline. Conclusion: This study found that although the ASIR and ASDR of stomach cancer have significantly declined, the absolute number of cases has continued to rise due to the continuous growth and aging of the population. In high SDI (socio-demographic index) regions, such as Western Europe and North America, the burden of stomach cancer has decreased. The high burden in low SDI regions needs to be alleviated through targeted interventions such as smoking control and improvement of dietary habits.

**Supplementary Information:**

The online version contains supplementary material available at 10.1186/s12889-025-23901-y.

## Introduction

Globally, stomach cancer remains a significant health challenge, consistently ranking among the leading causes of cancer-related deaths [1]. Due to the high mortality rate of stomach cancer, the long treatment time and the need for advanced medical interventions, as of 2017, the treatment cost of stomach cancer in China reached 23.84 billion CNY, ranking among the top three cancer inpatient expenses all year round. Further research found that the average hospitalization cost of stomach cancer was 19,876 US dollars, and the cost of radiotherapy and chemotherapy drugs accounted for the largest proportion [2, 3]. The treatment of stomach cancer imposes a substantial burden on society, making its prevention and management a pressing global health concern [4]. Over the past 30 years, enhanced public awareness of physical examinations and advancements in gastroscopy technology have led to improved detection methods, contributing to a decline in global incidence and mortality rates of stomach cancer. However, improvements have been inconsistent across regions, and significant differences remain between high-income and low - and middle-income countries [5]. The incidence of stomach cancer is multifactorial, involving the complex interaction of genetic, environmental and lifestyle factors [6–9]. Therefore, classifying and calculating the role of different risk factors is essential for developing effective prevention policies and optimizing resource allocation. The GBD study, a comprehensive and systematic analysis of global health trends, provides critical insights into the incidence, prevalence, and mortality associated with various diseases, including stomach cancer [10]. Importantly, the GBD framework incorporates the Socio-demographic Index (SDI), which is a comprehensive indicator reflecting the development level of a region based on per capita income, average educational attainment and total fertility rate. SDI can compare the disease burden in various regions at different stages of socio-economic development and is particularly useful in determining global health disparities. Utilizing the GBD database offers an invaluable opportunity to explore trends in stomach cancer over time, across different geographic regions, and among various demographic groups. By leveraging this extensive dataset, researchers can better understand the global and regional burden of stomach cancer, identify key risk factors, and evaluate the effectiveness of interventions. This study aims to analyze the GBD data to provide a detailed overview of the epidemiology of stomach cancer, with a focus on variations in incidence, death, and DALYs across different populations. Ultimately, such analyses could inform public health strategies and guide resource allocation to mitigate the global burden of stomach cancer.

## Method

### Overview

The methodologies employed in GBD 2021 have been detailed in previously published articles and are summarized here, with a primary focus on stomach cancer [11, 12]. Using the 10th edition of the International Classification of Diseases (ICD-10) as a guide, there are 29 malignant tumors cataloged within the GBD database. Stomach cancer encompasses all diagnoses coded as C16.0-C16.9 (stomach malignancies). As part of the health assessment process, this study adhered to guidelines for providing accurate and transparent information.

### Risk factor and SDI

Risk factor quantification is based on GBD 2021 comparative risk assessment [13]. According to the Global Burden of Disease Study Group, the Social-demographic Index (SDI) is a comprehensive measure of a country’s or region’s development based on per capita income, education level, and total fertility rate, which shows a strong correlation with health outcomes. The value of SDI ranges from 0 to 1. A higher value shows an advanced level of development [1]. 

### Statistics

This study is based on the GBD 2021 public database (http://ghdx.healthdata.org/gbd-results-tool) the sum of the secondary analysis of data. All data were standardized by the IHME (Institute for Health Metrics and Evaluation) team and included estimates of morbidity, mortality, DALYs and risk factors. The researchers did not directly process the raw data but extracted the calculated age standardization rate (ASR), EAPC and other indicators through the GBD result tool, and further carried out regional comparison and trend analysis.

Sequential patterns of stomach cancer incidence, DALYs, and mortality were quantified using Age-Standardized Rates (ASR), DALYs metrics, and Estimated Annual Percentage Changes (EAPCs). The ASR is calculated based on the GBD 2021 standard population, which incorporates a weighted average of the age distribution across regions of the world.

$$\mathrm{ASR}=\frac{\sum_{i=1}^A{\mathrm\alpha}_iw_i}{\sum_{i=1}^Aw_i}\times\;100,\;00$$

(αi: age-specific ratio for age group i; w: population count for corresponding age group i in the standard population; A: total number of age groups).

The Exponential Annualized Percentage Change (EAPC) serves as a statistical method to delineate data trends over time while forecasting future trajectories. EAPC estimates long-term trends by calculating an exponential representation of the annual rate of percentage change [14, 15]. An EAPC with its 95% Confidence Interval (CI) < 0 signifies a downward trend; conversely, smaller EAPC values indicate a more pronounced decline. Conversely, an EAPC with its 95% CI > 0 reflects an overall upward trend; larger EAPC values denote a more evident increase.

$$\mathrm y=\alpha+\beta x+\varepsilon\mathrm{EAPC}=100\ast\left(\exp\left(\mathrm\beta\right)-1\right)$$

Based on the GBD 2021 comparative risk assessment framework, the contribution of smoking, high-salt diet and other factors to stomach cancer DALYs was quantified by population attribution score (PAF). The association between SDI and disease burden was performed using a piecewise linear regression model, with trends fitted by SDI quintile grouping.

### BAPC

We employed the BAPC (Bayesian age-period-cohort) model to forecast the Age-Standardized Incidence Rate (ASIR) and Age-Standardized Death Rate (ASDR) of stomach cancer over the next two decades. BAPC model is a statistical model that combines data from three-time dimensions: age, period and cohort. Among them, the age dimension reflects the disease risk of different age groups. The period dimension reflects the change of disease incidence in different time periods. The cohort dimension considers the evolution of disease risk over the life cycle of a population born at the same time. Through the combination of these three dimensions, the BAPC model can capture the epidemic trends and patterns of diseases more comprehensively. The BAPC model is a robust statistical framework for time series forecasting, capable of handling non-stationary data while enhancing prediction accuracy through autoregressive and moving average components [16, 17]. Consequently, it serves as an effective predictor for future trends in the burden of stomach cancer.

### UI

Uncertainty interval (UI) ‌ refers to the uncertainty range of the forecast result caused by various uncertain factors in the forecasting process. This uncertainty interval can help us better understand the possible range of predicted outcomes and make more rational decisions. GBD 2021 computes UI through 1,000 iterations of Monte Carlo simulation, reflecting the combined effects of data inputs, model parameters, and sampling errors. All estimates reported in this study are presented in a median and 95% UI.

### Role of the funding source

The funders of the research have no role in report design, data collection, data analysis, data interpretation, or paper writing.

## Results

### The incidence burden of stomach cancer

**Fig. 1:**
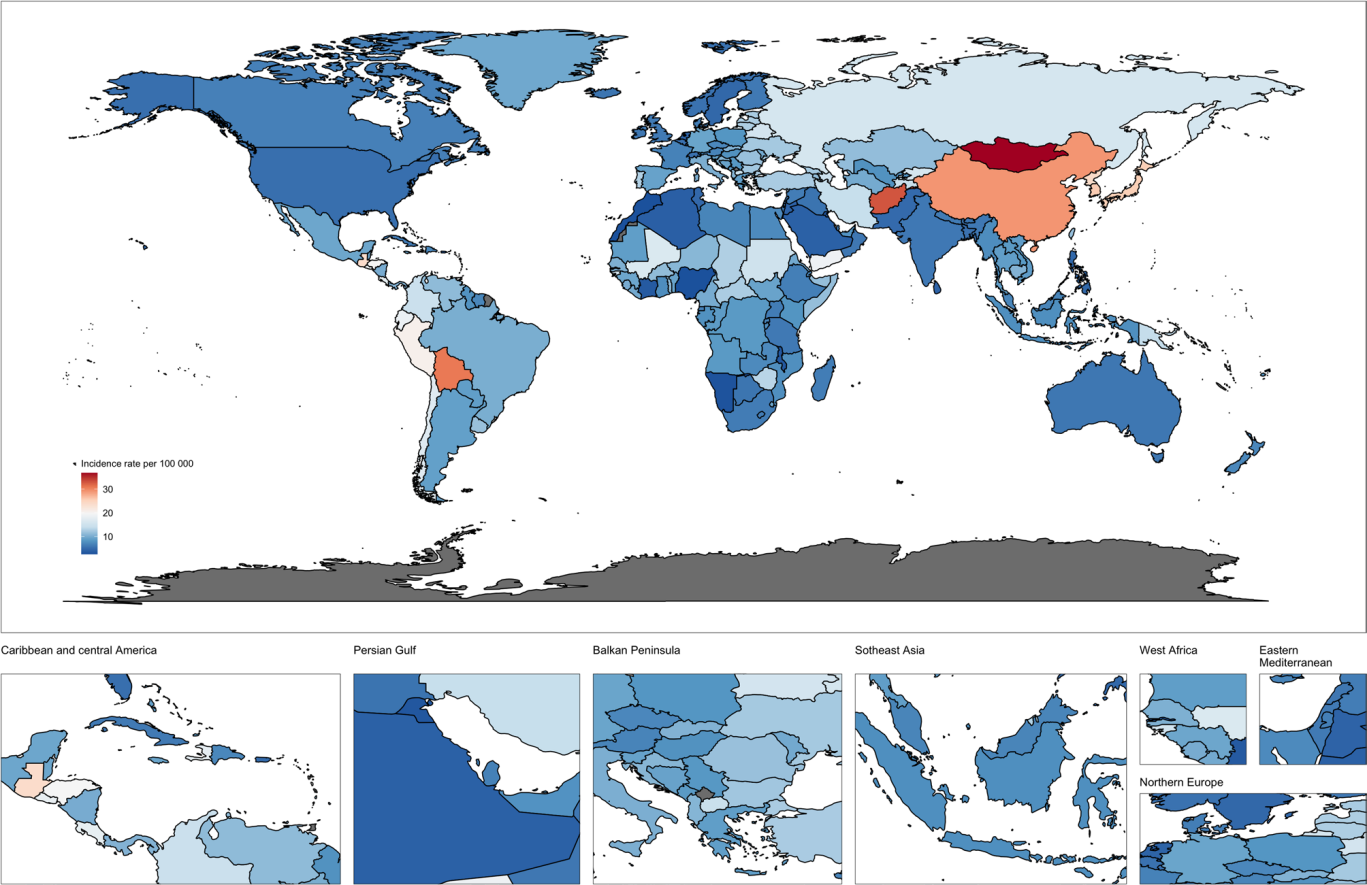
Age-standardized incidence of stomach cancer in both sexes combined in 204 countries around the world

Globally, the incidence number of stomach cancer rose from 980,899 cases in 1990(Table 1) (95% UI 891307,1072236) to 1,230,233 cases in 2021 (95% UI: 1052350, 1409970) (Fig. 1; Table 2). The ASIR decreased from 24.76 per100,000 population in 1990 to 14.33 per100,000 population in 2021, with higher ASIR in males than females (Figure S3). The EAPC of global ASIR from 1990 to 2021 was − 1.81% (95% CI: −1.87 to −1.75), indicating an average annual decline rate of 1.81%, but there were significant differences among regions. Segmented regression showed that the decline in ASIR in high SDI regions accelerated after 2000 (EAPC: −2.3%, 95% CI: −2.5 to −2.1), while in low SDI regions, the decline slowed down after 2010 (EAPC: −0.9%, 95% CI: −1.1 to −0.7) (Table S1). In terms of sex and age, the incidence number of global stomach cancer in 2021 was predominantly observed in individuals over the age of 40, with the highest prevalence occurring within the 70–74 age group. Notably, the incidence among men significantly exceeded that of women. Compared to 1990, there was a marked increase in stomach cancer cases among those aged 65–74 and 85–89 in 2021, while a decline was noted in the 15–29 age group; other age groups remained relatively stable (Fig. S2). The ASIR of the seven super regions(Central Europe, Eastern Europe, and Central Asia; High-income; Latin America and Caribbean; North Africa and Middle East; South Asia; Southeast Asia, East Asia, and Oceania; Sub-Saharan Africa) also varied widely, with Southeast Asia, East Asia, and Oceania having the highest ASIR at 23.76 per100,000 population (95% UI: 18.70, 29.22), while South Asia was the lowest, at 5.70(95% UI:4.92, 6.84) per100,000 population. While there are also differences between the five SDI regions (Table 2). The highest ASIR was 19.62 per100,000 population people in the High-middle SDI region (95% UI: 16.02, 23.13) and the lowest was 7.68 in the Low-middle SDI region (95% UI: 6.71, 8.77) per100,000 population people. Regionally, between 1990 and 2021, there were variations in incidence across the 21 Global Burden of Disease (GBD) regions, with East Asia exhibiting the highest ASIR at 28.64 per100,000 population people (95% UI: 22.23, 35.55). In contrast, High-Income North America reported a lower ASIR of 5.23 per100,000 population people (95% UI: 4.90, 5.46) (Table 2). This is significantly related to the current situation of high helicobacter pylori infection in East Asia and low helicobacter pylori infection in high-income North America. Furthermore, Western Sub-Saharan Africa experienced the smallest decrease in incidence rate over this period (EAPC: −0.37; 95% CI: −0.45 to −0.30), The biggest decline was recorded in North America’s High-Income Sector (EAPC: −3.08; 95% CI: −3.15 to −3.01). (Table S1)

**Table 1:** Cases of deaths, incidence and DALYs of stomach cancer in 1990, and age-standardized rates by GBD region

	**Incidence**		Death		DALYs (Disability-Adjusted Life Years)	
	number	Age-standardized incidence rate	number	Age-standardized death rate	numbers	Age-standardized DALYs rate
Global	980,899 (891307,1072236)	24.76 (22.58,27)	854,185 (772885,939973)	22.01 (20.03,24.19)	23,237,292 (20605349,25526194)	559.72 (499.09,615.77)
21 GBD regions						
Andean Latin America	6287 (5533,7155)	31.13 (27.43,35.5)	6455 (5696,7324)	32.8 (28.93,37.32)	168,541 (147325,190607)	773.16 (678.85,875.03)
Australasia	2409 (2259,2538)	10.26 (9.63,10.83)	1804 (1690,1906)	7.73 (7.25,8.16)	39,867 (37637,41910)	171.48 (161.92,180.59)
Caribbean	3132 (2870,3439)	12.15 (11.12,13.32)	3138 (2873,3450)	12.38 (11.34,13.57)	79,115 (71374,87961)	296.37 (268.46,329.4)
Central Asia	12,594 (11989,13286)	26.36 (25.05,27.82)	12,366 (11762,13059)	26.28 (24.93,27.77)	359,741 (343480,378737)	720.77 (687.22,760.05)
Central Europe	27,272 (26183,28244)	18.34 (17.57,19.01)	27,224 (26133,28216)	18.5 (17.72,19.19)	666,468 (641593,689338)	444.41 (427.88,459.51)
Central Latin America	16,300 (15735,16774)	20.19 (19.33,20.81)	16,459 (15832,16952)	21.14 (20.17,21.8)	427,467 (414311,439042)	482.23 (466.65,496.63)
Central Sub-Saharan Africa	2568 (1894,3121)	11.51 (8.58,13.89)	2570 (1888,3136)	12.13 (9.06,14.67)	77,401 (57088,94838)	305.95 (225.6,372.66)
East Asia	415,521 (345214,486249)	47.14 (39.54,55.52)	381,435 (318259,449024)	45.18 (38.27,53.36)	10,985,613 (9060114,12871606)	1160.15 (965.28,1363)
Eastern Europe	92,743 (90216,94582)	32.94 (31.98,33.58)	83,348 (81061,85100)	29.71 (28.85,30.32)	2,264,040 (2215067,2307995)	805.45 (787.5,821.16)
Eastern Sub-Saharan Africa	8228 (6526,9433)	10.65 (8.47,12.15)	8233 (6531,9437)	11.09 (8.81,12.7)	252,008 (199160,287489)	292.75 (232.08,335.22)
High-income Asia Pacific	130,992 (123589,135804)	65.16 (61.41,67.66)	72,724 (67979,75561)	37.06 (34.46,38.6)	1,831,592 (1693439,1906727)	900.48 (832.19,937.87)
High-income North America	29,244 (27507,30219)	8.3 (7.84,8.56)	19,836 (18496,20571)	5.57 (5.21,5.77)	438,698 (419948,450664)	129.31 (124.2,132.72)
North Africa and Middle East	24,709 (18612,27925)	14.71 (11.07,16.6)	24,484 (18566,27726)	15.19 (11.51,17.15)	707,934 (531826,799708)	380.2 (288.74,429.88)
Oceania	513 (364,662)	17.12 (12.57,21.85)	497 (358,641)	17.76 (13.08,22.53)	15,969 (11130,20718)	456.41 (329.1,588.19)
South Asia	46,713 (39946,59247)	7.71 (6.59,9.93)	46,010 (39093,58610)	7.88 (6.68,10.17)	1,432,660 (1232646,1789557)	215.57 (183.72,273.18)
Southeast Asia	28,024 (22597,32063)	10.72 (8.69,12.28)	27,448 (22187,31331)	10.92 (8.91,12.54)	820,413 (659024,941613)	285.4 (229.89,326.55)
Southern Latin America	8563 (8077,9055)	18.7 (17.61,19.79)	8435 (7955,8911)	18.65 (17.56,19.75)	201,215 (190126,212735)	432.39 (408.29,457.03)
Southern Sub-Saharan Africa	2178 (1715,2438)	7.92 (6.21,8.87)	2151 (1697,2411)	8.13 (6.38,9.11)	62,836 (49869,70324)	208.13 (164.69,233.47)
Tropical Latin America	\	18.91 (17.91,19.68)	16,803 (16029,17446)	19.54 (18.45,20.37)	449,287 (432709,464461)	463.86 (444.88,480.64)
Western Europe	99,469 (93628,102944)	16.99 (16.04,17.56)	86,059 (80686,89117)	14.52 (13.64,15.03)	1,769,700 (1688901,1823382)	316.11 (302.86,324.92)
Western Sub-Saharan Africa	6579 (5585,7728)	7.58 (6.45,8.96)	6705 (5699,7866)	8.01 (6.85,9.5)	186,729 (157190,218063)	196.52 (166.83,230.65)
7 GBD super regions						
Central Europe, Eastern Europe, and Central Asia	132,609 (129231,135222)	27.69 (26.91,28.25)	122,937 (119629,125526)	25.85 (25.12,26.41)	3,290,250 (3223174,3354273)	683.77 (669.77,697.28)
High-income	270,676 (257676,278214)	22.75 (21.69,23.39)	188,858 (178287,194603)	15.69 (14.83,16.17)	4,281,072 (4098605,4389855)	369.72 (353.82,379.02)
Latin America and Caribbean	42,582 (40960,44022)	19.72 (18.91,20.4)	42,856 (41171,44291)	20.52 (19.59,21.26)	1,124,409 (1085651,1159449)	479.98 (462.26,495.82)
North Africa and Middle East	24,709 (18612,27925)	14.71 (11.07,16.6)	24,484 (18566,27726)	15.19 (11.51,17.15)	707,934 (531826,799708)	380.2 (288.74,429.88)
South Asia	46,713 (39946,59247)	7.71 (6.59,9.93)	46,010 (39093,58610)	7.88 (6.68,10.17)	1,432,660 (1232646,1789557)	215.57 (183.72,273.18)
Southeast Asia, East Asia, and Oceania	444,057 (368488,518050)	38.63 (32.5,45.08)	409,380 (342477,479101)	37.07 (31.34,43.66)	11,821,994 (9743859,13743522)	956.91 (795.58,1117.85)
Sub-Saharan Africa	19,553 (15886,22050)	\	19,659 (16082,22155)	9.55 (7.89,10.78)	578,973 (470313,650330)	244.16 (198.67,274.97)
5 SDI quintiles regions						
High SDI	381.13 (362.21,392.23)	23.13 (22.05,23.8)	175,683 (166255,181197)	15.86 (15.01,16.37)	4,099,422 (3902053,4218897)	381.13 (362.21,392.23)
High-middle SDI	802.75 (711.79,876.76)	33.33 (30.09,36.1)	304,773 (274911,330960)	31.08 (28.09,33.7)	8,241,238 (7281203,9010822)	802.75 (711.79,876.76)
Low SDI	311.98 (248.62,357.31)	11.41 (9.04,13.03)	26,410 (21016,30213)	11.9 (9.41,13.57)	795,640 (636078,910967)	311.98 (248.62,357.31)
Low-middle SDI	274.45 (239.89,333.49)	10.14 (8.98,12.42)	62,598 (54993,76278)	10.45 (9.25,12.79)	1,874,805 (1629366,2261562)	274.45 (239.89,333.49)
Middle SDI	721.81 (624.06,835.38)	28.89 (25.13,33.44)	284,016 (246760,329474)	28.1 (24.61,32.65)	8,208,596 (7034443,9506074)	721.81 (624.06,835.38)

**Table 2:** Cases of deaths, incidence and DALYs of stomach cancer in 2021, and age-standardized rates by GBD region

	Incidence		Death		DALYs (Disability-Adjusted Life Years)	
	number	Age standardized incidence rate (per 100000 population)	number	Age standardized death rate (per 100000 population)	numbers	Age standardized DALYs rate (per 100000 population)
Global	1,230,233 (1052350,1409970)	14.33 (12.23,16.41)	954,374 (821751,1089577)	11.2 (9.62,12.73)	22,786,633 (19576344,26118869)	262.75 (226.08,301.02)
21 GBD regions						
Andean Latin America	12,593 (10226,15421)	21.48 (17.41,26.27)	12,368 (10039,15114)	21.33 (17.3,26.08)	292,675 (235472,359119)	485.41 (391.23,595.07)
Australasia	3187 (2823,3478)	5.93 (5.32,6.43)	2191 (1921,2388)	3.91 (3.49,4.25)	42,612 (38796,46055)	84.88 (77.89,91.35)
Caribbean	4279 (3708,4900)	7.97 (6.91,9.13)	4191 (3626,4808)	7.79 (6.74,8.94)	104,402 (89158,120825)	195.67 (167.08,226.69)
Central Asia	9763 (8797,10942)	11.78 (10.66,13.11)	9332 (8400,10425)	11.56 (10.46,12.83)	264,445 (236310,298863)	298.62 (268.02,336.43)
Central Europe	20,269 (18700,21903)	9.19 (8.49,9.94)	19,182 (17694,20668)	8.54 (7.89,9.22)	418,848 (387111,452190)	201.3 (185.93,217.26)
Central Latin America	29,824 (26593,33520)	11.98 (10.69,13.44)	28,763 (25594,32289)	11.69 (10.41,13.09)	722,803 (644910,813721)	282.56 (252.21,317.88)
Central Sub-Saharan Africa	4664 (3482,5777)	8.54 (6.39,10.51)	4607 (3474,5734)	8.93 (6.76,11.06)	139,267 (104199,175157)	220.04 (165.52,273.38)
East Asia	624,688 (483569,778629)	28.64 (22.23,35.55)	455,947 (355895,567156)	21.26 (16.59,26.22)	10,921,589 (8491323,13670612)	496.94 (386.19,619.66)
Eastern Europe	51,006 (46933,54984)	14.67 (13.48,15.85)	42,700 (39316,46074)	12.15 (11.17,13.12)	1,031,465 (943209,1119000)	306.55 (280.01,332.59)
Eastern Sub-Saharan Africa	11,642 (9555,13652)	6.8 (5.65,7.95)	11,542 (9518,13490)	7.09 (5.92,8.28)	344,564 (281270,404252)	175.48 (144.38,205.24)
High-income Asia Pacific	123,451 (107133,133459)	25.43 (22.8,27.27)	70,689 (60268,77102)	13.13 (11.52,14.14)	1,194,021 (1061152,1287243)	273.07 (250.22,294.45)
High-income North America	33,113 (30756,34704)	5.23 (4.9,5.46)	19,363 (17658,20393)	2.95 (2.72,3.1)	419,798 (396159,437428)	70.88 (67.42,73.54)
North Africa and Middle East	42,569 (29749,48466)	9.67 (6.82,10.95)	39,717 (27883,45110)	9.43 (6.7,10.67)	1,057,332 (732444,1215400)	217.41 (151.81,247.77)
Oceania	1009 (787,1258)	13.26 (10.49,16.34)	957 (744,1201)	13.45 (10.67,16.61)	30,296 (23279,38275)	341.81 (265.8,428.05)
Southeast Asia	47,761 (41101,56374)	7.27 (6.28,8.69)	43,303 (37510,51160)	6.83 (5.94,8.16)	1,193,413 (1021566,1404696)	170.78 (146.62,201.09)
Southern Latin America	9965 (9007,10841)	11.39 (10.32,12.38)	9292 (8379,10092)	10.51 (9.47,11.41)	205,763 (188547,223240)	241.14 (221.13,261.57)
Southern Sub-Saharan Africa	4055 (3376,4570)	7.02 (5.8,7.86)	3967 (3307,4472)	7.13 (5.92,7.98)	111,785 (94036,128129)	177.66 (148.51,201.91)
Tropical Latin America	25,611 (23899,26903)	9.99 (9.3,10.51)	24,867 (23070,26204)	9.78 (9.05,10.32)	619,369 (586946,648930)	237.81 (225.12,249.26)
Western Europe	73,911 (65944,78835)	7.81 (7.11,8.25)	56,322 (49569,60192)	5.57 (5.02,5.9)	1,038,057 (950524,1095124)	120.38 (112.31,126.18)
Western Sub-Saharan Africa	12,070 (9336,14239)	6.33 (5.01,7.47)	12,151 (9468,14304)	6.69 (5.34,7.9)	338,256 (257059,405471)	156.08 (120.91,184.46)
7 GBD super regions						
Central Europe, Eastern Europe, and Central Asia	81,038 (75806,85462)	12.52 (11.72,13.2)	71,214 (66787,75009)	10.92 (10.24,11.51)	1,714,757 (1609234,1809234)	273.14 (256.07,288.17)
High-income	243,627 (216524,258960)	10.84 (9.85,11.41)	157,857 (138007,168385)	6.62 (5.92,7)	2,900,250 (2645936,3052300)	140.88 (130.98,147.1)
Latin America and Caribbean	72,306 (65878,79212)	11.71 (10.66,12.84)	70,188 (63894,76939)	11.47 (10.43,12.57)	1,739,249 (1592169,1904520)	276.2 (252.78,302.5)
North Africa and Middle East	42,569 (29749,48466)	9.67 (6.82,10.95)	39,717 (27883,45110)	9.43 (6.7,10.67)	1,057,332 (732444,1215400)	217.41 (151.81,247.77)
South Asia	84,803 (73745,102263)	5.7 (4.92,6.84)	82,924 (71235,99367)	5.72 (4.91,6.84)	2,295,875 (2004284,2773948)	145.55 (126.31,175.43)
Southeast Asia, East Asia, and Oceania	673,458 (529941,832946)	23.76 (18.7,29.22)	500,207 (397438,614535)	18.09 (14.29,22.1)	12,145,298 (9629467,15076227)	420.05 (333.9,519.39)
Sub-Saharan Africa	32,431 (26011,37310)	6.82 (5.56,7.81)	32,266 (25949,37054)	7.13 (5.85,8.16)	933,872 (743843,1085482)	172.69 (138.38,198.9)
5 SDI quintiles regions						
High SDI	239,119 (215071,256938)	11.16 (10.21,11.91)	153,539 (136670,165354)	6.83 (6.18,7.34)	2,897,404 (2654584,3101177)	146.1 (135.56,155.89)
High-middle SDI	387,196 (315957,457286)	19.62 (16.02,23.13)	295,105 (244787,343611)	14.93 (12.4,17.36)	6,901,607 (5703610,8141888)	353.18 (291.89,416.78)
Low SDI	41,388 (32600,46956)	8.13 (6.44,9.22)	41,192 (32515,46864)	8.46 (6.71,9.6)	1,198,903 (940407,1371290)	209.77 (165.6,238.95)
Low-middle SDI	110,396 (97098,126222)	7.68 (6.71,8.77)	107,456 (94064,122211)	7.71 (6.69,8.76)	2,953,125 (2599859,3357038)	192.56 (169.42,219.14)
Middle SDI	451,465 (368224,542292)	16.91 (13.79,20.28)	356,459 (294160,423770)	13.72 (11.31,16.22)	8,820,717 (7336337,10567624)	320.24 (266.07,382.87)

### The death burden of stomach cancer

**Fig. 2:**
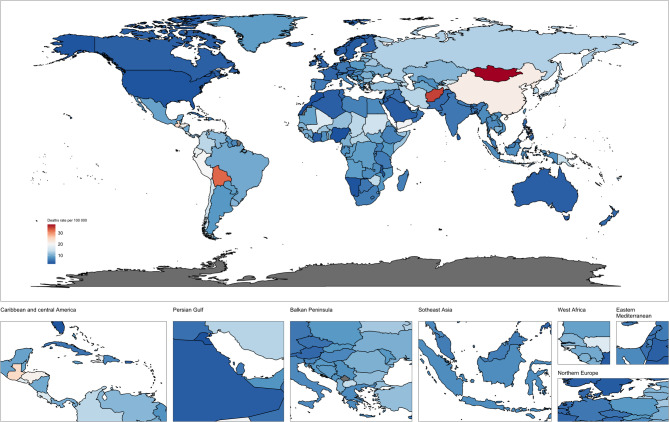
Age-standardized death rate of stomach cancer in both sexes combined in 204 countries around the world

The number of global deaths in 2021 was 954374 (95% UI: 821751, 1089577) (Table 2) and the ASDR was 11.20 per100,000 population (95% UI: 9.62, 12.73), reflecting a reduction of 10.8 per 100,000 population since 1990 (Fig. 2, Fig.S3). Deaths have risen overall, particularly among men, though the number of deaths for those under 64 has decreased since 1990. In contrast, deaths in those over 65 have increased, with the sharpest rise observed in men aged 80–89 and women aged 85–89, likely due to higher life expectancy (Fig. S2). Of the seven super regions, South-East Asia, East Asia and Oceania had the highest number of stomach cancer deaths (combined 500,207 cases, 95% UI: 397,438–614,535), while sub-Saharan Africa had the lowest number of deaths (32,266 cases, 95% UI: 25,949 − 37,054).In the five SDI regions, the Medium-High SDI region recorded the highest ASDR (14.93, 95% UI: 12.40, 17.36) and the highest age-standardized DALY rate (802.75, 95% UI: 711.79 to 876.76). Both ASDR and DALY rates have declined across all SDI regions, with the largest decreases seen in high SDI regions: a reduction of 2.79 (95% CI: −2.81 to −2.76) for ASDR and 3.15 (95% CI: −3.18 to −3.13) for DALYs. Among the 21 GBD regions, East Asia recorded the highest number of stomach cancer deaths in 2021, with 455947 cases (95% UI: 355895,567616), likely influenced by factors such as dietary habits and Helicobacter pylori infections. As expected, East Asia also had the highest (ASDR at 21.26 per100,000 population (95% UI: 16.59–26.22). In contrast, High-income North America had the lowest ASDR, at 2.95 per100,000 population (95% UI: 2.72–3.10). At the national level, China had the highest number of stomach cancer deaths in 2021, with 445012 cases (95% UI: 344736,555833), largely due to its large population (Table 2). China’s ASDR was 21.51 per100,000 population (95% UI: 16.66–26.61), slightly above the regional average for East Asia. The EAPC in ASDR for China was − 3.5 (95% CI: −4.26 to −2.72), indicating a decline greater than the global average, reflecting the country’s efforts to reduce stomach cancer mortality. Meanwhile, countries like Tokelau, Niue, and Nauru had the fewest stomach cancer deaths. (Fig. 2, Table S1)

### The dalys burden of stomach cancer

**Fig. 3:**
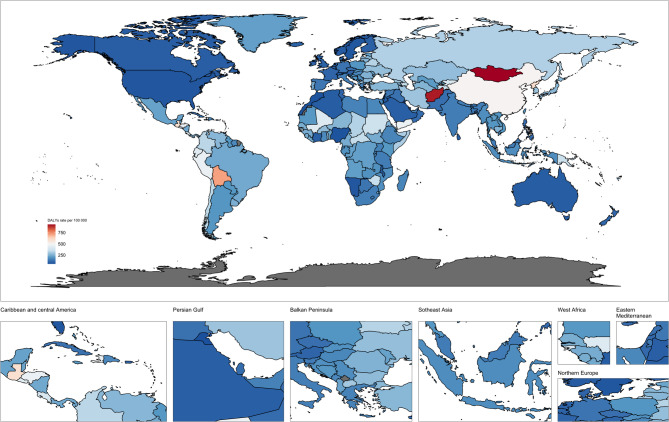
Age-standardized DALYs rate of stomach cancer in both sexes combined in 204 countries around the world. DALYs, disability-adjusted life–years

In 2021, the global disability-adjusted life years (DALYs) due to stomach cancer amounted to 22,786,633 (95% UI: 19576344,26118869) (Table 2), with an age-standardized DALYs rate of 262.75 per100,000 population people (95% UI: 226.08-301.02), reflecting a decrease of 336.98 from 1990 to 2021 (Fig. 3, Fig.S3). The decline in DALYs largely mirrors the reduction in deaths, primarily affecting individuals under 64, with the steepest drop observed among men and women aged 60–64. However, DALYs increased in people over 65, especially among males aged 70–74 and females aged 85–89 (Fig. S2). In the five SDI regions, the Middle SDI region had the highest DALY count at 8,820,717(95% UI: 9629467,15076227), while the Low SDI region had the lowest at 1,198,903 (95% UI: 940406,1371290). All SDI regions experienced a decline in the age-standardized DALY rate, with the highest rate of 353.18 per100,000 population (95% UI: 291.89,416.78) in the High-middle SDI region, and the lowest rate of 192.56 per 100,000 population (95% UI: 169.43,219.14) in the Low-middle SDI region. Of the 21 GBD regions, Southeast Asia, East Asia, and Oceania reported the most DALYs, amounting to 12145298.36 (95% UI: 9629467.25,15076227.15). Between 1990 and 2021, age-standardized DALY rates declined in every region. East Asia exhibited the highest rate at 496.94 per 100,000 population (95% UI: 386.19–619.66) (Table 2). The greatest decrease was observed in the High-income Asia Pacific region, with an estimated annual percentage change (EAPC) of −3.91 (95% CI: −3.95 to −3.86), whereas Southern Sub-Saharan Africa experienced the smallest decline (EAPC of −0.5; 95% CI: −0.84 to −0.15) (Table S1). In 2021, China recorded 10,642,127 stomach cancer DALYs (95% UI: 8222106.35–13383779.05), while Mongolia had the highest age-standardized DALY rate at 930.44 per 100,000 population (95% UI: 747.52–1,157.92) (Fig. 3).

### Risk factors

**Fig. 4:**
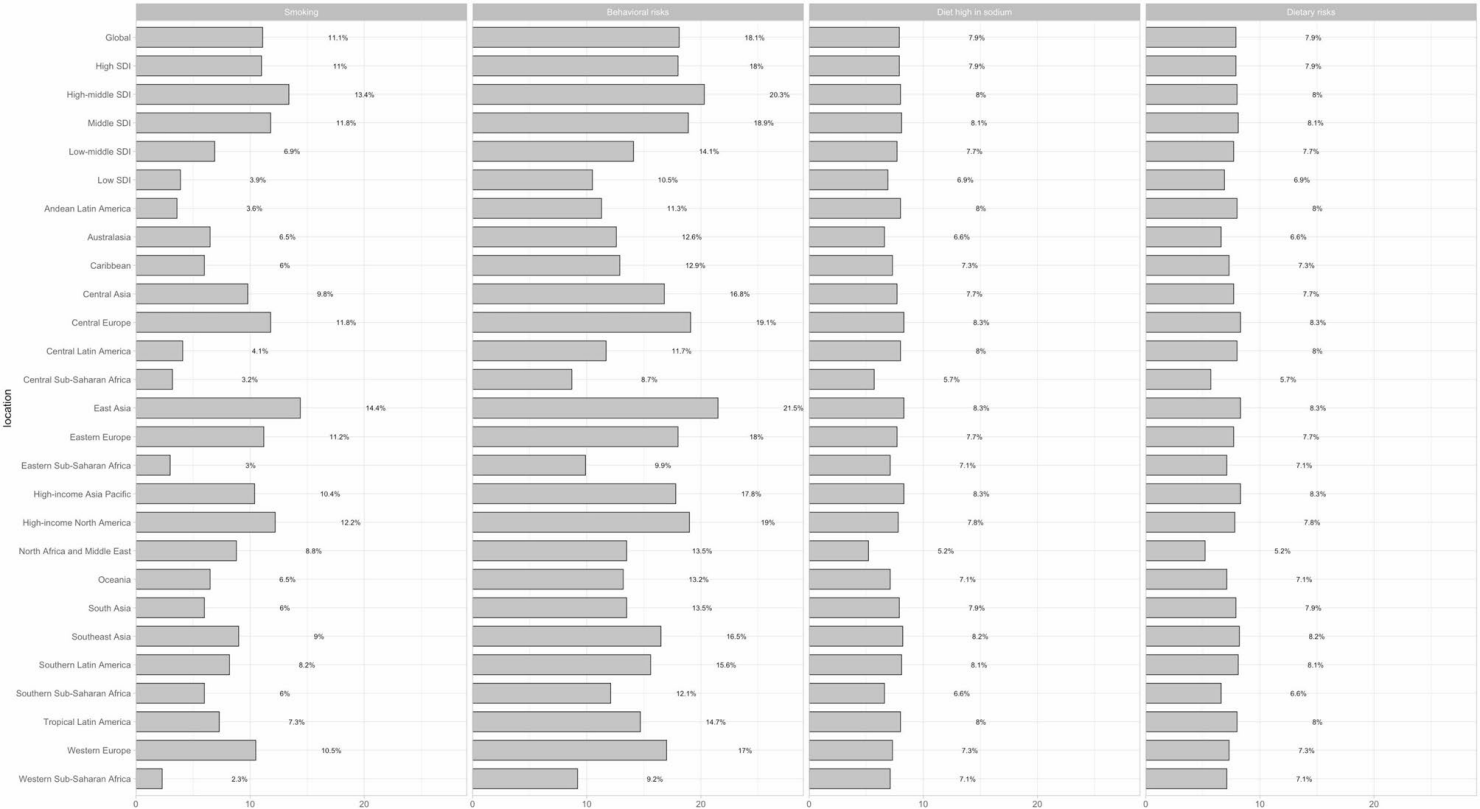
Percentage of stomach cancer age-standardized DALYs attributable to high-sodium diet, behavioral risks, smoking and diet risks in 2021, globally and for 21 GBD regions and 5 SDI regions

We next examined various risk factors contributing to DALYs, focusing primarily on the impact of smoking and high-sodium diets (Fig. 4), though Helicobacter pylori infection remains a leading global cause of stomach cancer. Figure 4 illustrates the global and regional distribution of indicators associated with smoking, behavioral risks, high-sodium diets, and dietary risks in population health. The prevalence of smoking is highest in East Asia at 14.4%, and lowest in Western Sub-Saharan Africa at 2.3%. Other regions, including Central Europe and High-income North America, also exhibit relatively high percentages (11.8% and 13.2%, respectively). Globally, the average level of behavioral risk is elevated, with the highest risk observed in high- and middle-income SDI regions (20.3%) and the lowest in Central Sub-Saharan Africa (8.7%). The risk associated with a high-sodium diet is relatively uniform across most regions, ranging from 6 to 8%. Central and Eastern Europe report a slightly higher risk at 8.3%. The global average dietary risk aligns closely with that of a high-sodium diet, typically falling between 7% and 8%, with Central and Eastern Europe again showing a marginally higher risk ratio (8.3%). Overall, high- and middle-income regions, such as High-income North America and East Asia, exhibit higher rates of smoking and behavioral risks. Conversely, low-income regions like Sub-Saharan Africa demonstrate lower smoking and behavioral risks but still experience a notable proportion of diet-related risks.

### Patterns of change in different SDI levels and baseline burden

**Fig. 5:**
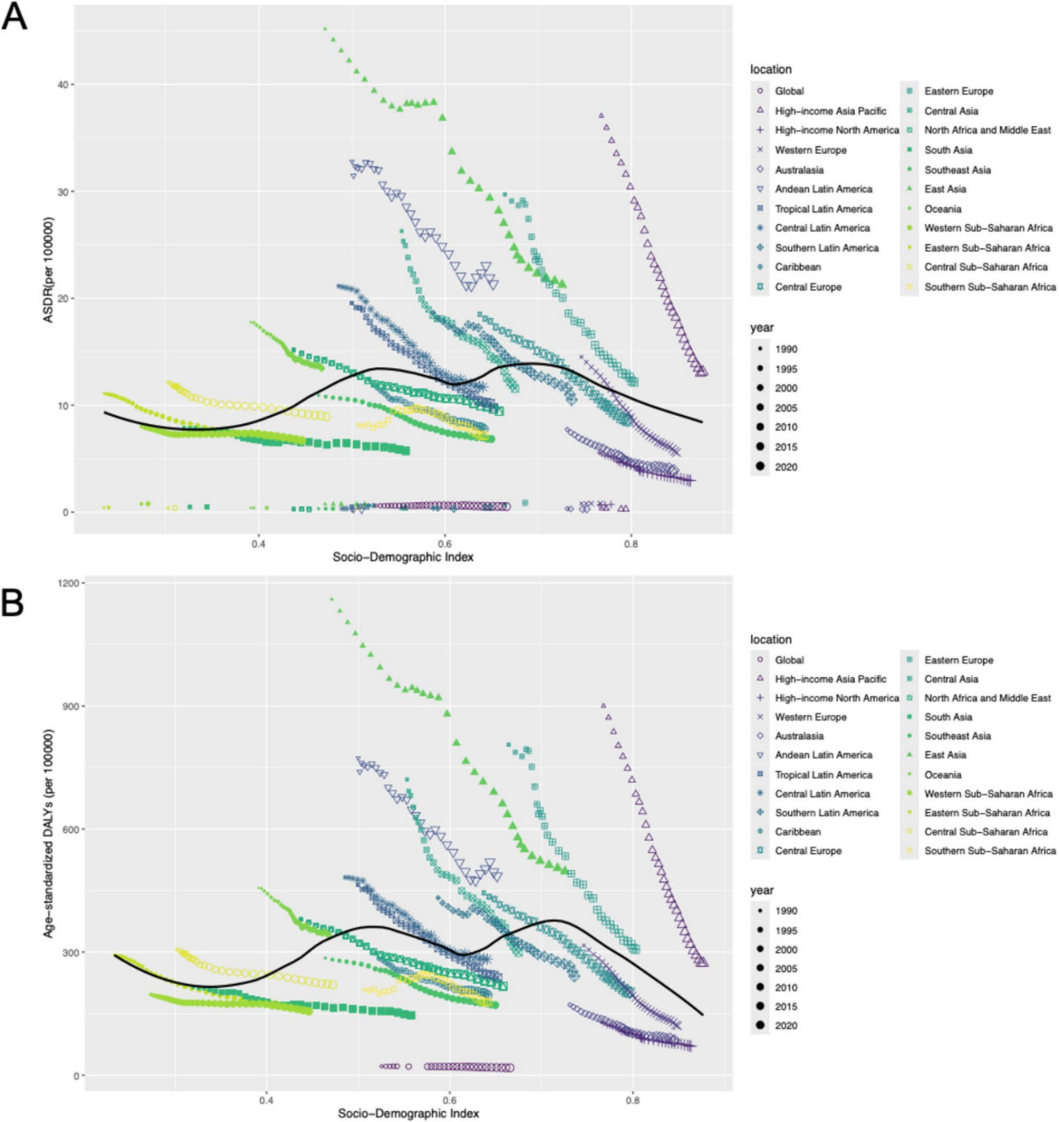
Age-standardized burden rate attributable to stomach cancer across 21 GBD regions by socio-demographic index for both sexes, 1990–2021. (A) ASDR; (B) age-standardized disability-adjusted life year rate. The black line was an adaptive association fitted with adaptive Loess regression based on all data points. GBD, Global Burden of Disease Study

It can be seen from the Figure that ASDR is roughly M-shaped (Fig. 5), when the SDI is less than 0.5, the above three indicators of ASDR gradually increase, when the SDI is in the range of 0.5–0.7, it gradually decreases and then gradually rises to a V-shape, and when the SDI is greater than 0.7, the above indicators decrease rapidly. The relationship between DALYs and SDI is like to ASDR (Fig. 5). Overall, regions with higher SDI (right side of the Figure) have lower mortality rates, while regions with lower SDI (left side of the Figure) have higher mortality rates. Overall, mortality rates decreased in most regions from 1990 to 2020, but the magnitude of the decline varied from region to region. It can also be observed that mortality rates are relatively high in low-SDI regions such as sub-Saharan South Africa, while mortality rates are relatively low in high-income countries and regions (Western Europe, North America). In low-SDI areas (sub-Saharan Africa, South Africa, South Asia), health losses are more severe and DALYs are higher. High SDI areas (North America, Europe, East Asia) have relatively small health losses and lower DALYs. Over time (from 1990 to 2020), DALYs decreased in most regions, especially in the middle and high SDI regions.

### Future and forecast

We have also developed global projections for the ASIR, ASDR and Age-Standardized DALYs Rate for stomach cancer. The global ASIR is projected to reach 12.35 per 100,000 population by 2030. The ASIR for men is expected to be 17.69 per 100,000, while for women it will be 8.55 per 100,000. As historically observed, the ASIR for women has consistently been lower than that for men. However, with global population growth, the absolute incidence of stomach cancer is anticipated to increase. Furthermore, the global ASDR is projected to decrease to 9.56 per 100,000 by 2030, with the ASDR for men reaching 12.75 per 100,000. For women, the ASDR is projected to be 5.96 per 100,000. These year-on-year declines in ASDR reflect the success of global public health efforts to reduce cancer-related mortality.It is estimated that by 2030, the ASR of female stomach cancer DALYs will be 142.34 cases per 100,000; the ASR of stomach cancer DALYs in male will be 300.79 cases per 100,000 (Fig. S1).

## Discussion

According to the 2021 GBD data, stomach cancer continues to be a major contributor to global cancer incidence and mortality. Nearly one million new stomach cancer cases are diagnosed annually, with men showing significantly higher incidence rates than women [18]. Geographically, East Asia—specifically China, Japan, and South Korea—reports the highest rates of stomach cancer, with other high-risk regions including parts of Latin America and Eastern Europe [19–22]. Stomach cancer mortality rates are particularly high in low- and middle-income countries, largely due to delayed diagnoses and limited access to treatment. In contrast, high-income countries have experienced a significant decline in mortality rates, largely attributable to early detection and advancements in treatment methods [23]. Stomach cancer incidence increases sharply with age, particularly after the age of 50, and remains relatively rare in individuals under 40. However, since 1990, there has been a rise in stomach cancer cases among younger individuals, likely driven by shifts in modern lifestyle, dietary habits, and elevated work-related stress [24–26]. The incidence is approximately twice as common in men as in women, a disparity likely associated with lifestyle factors such as smoking, alcohol consumption, Helicobacter pylori infection, and work-related stress [24]. 

Despite a decline in global age-standardized rates, stomach cancer remains the fifth most common cancer and the fifth leading cause of cancer-related deaths worldwide, as of 2021, according to the World Health Organization. While this marks an improvement from 2019, the overall burden on health systems in high-risk regions remains substantial. Factors such as population growth and aging contribute to the continued rise in absolute numbers of cases and deaths in many regions, despite improvements in relative rates. DALYs related to stomach cancer are particularly high in East Asia, South Asia, and certain Eastern European countries [10]. The elevated incidence and mortality rates in these regions have made stomach cancer a critical local public health issue. Should the incidence in East Asia continue to decrease, a corresponding reduction in cases and deaths can be expected. Consequently, future efforts in stomach cancer prevention and treatment should be concentrated in East Asia. Despite Japan’s high incidence of stomach cancer, patient survival rates are notably high, primarily due to nationwide screening programs initiated by the government in the 1980s. Approximately 6 million individuals participate in these screenings annually [27]. This extensive screening has resulted in over 50% of cases being diagnosed at an early stage, with early detection and treatment rates as high as 70%. In addition to early diagnosis, Japan is a global leader in endoscopic treatment. Endoscopic resection (ER) is now the standard treatment for early-stage stomach cancer. Japan has also pioneered laparoscopic surgery techniques, enabling procedures through small incisions, which minimizes trauma and enhances patient quality of life. Japanese stomach cancer treatment emphasizes individualized and precise care, with treatment plans tailored to each patient’s specific condition. In the future, stomach cancer treatment in Japan is expected to advance further towards minimally invasive techniques, stomach preservation, functional preservation, and personalized care [28–31]. Other countries can learn from Japan’s experience in stomach cancer prevention and treatment, adapting these strategies to their specific contexts to reduce the global public health burden.

The primary risk factors for stomach cancer have been extensively studied. Helicobacter pylori (Hp) infection is widely regarded as the most important pathogenic factor of stomach cancer. Hp infection can lead to chronic gastritis, atrophic gastritis and atypical hyperplasia of stomach mucosa, which significantly increases the risk of stomach cancer [32]. Data from 2021 show that Hp infection accounts for the largest proportion of the global stomach cancer burden, with up to more than 80% of non-cardia stomach cancer cases associated with Hp infection [33]. Therefore, Hp screening and eradication therapy are key to the global strategy for the prevention and control of stomach cancer. This study found that regional differences in stomach cancer burden were consistent with the epidemiological characteristics of Hp infection. For example, high infection rates in East Asia directly drive its high age-standardized incidence (ASIR), while low infection rates in North America and Western Europe are associated with low incidence. Globally, the geographic distribution of Hp infection rates was positively associated with stomach cancer incidence. In East Asia, China, Korea, and Japan have higher Hp infection rates, and the ASIR of stomach cancer in these countries is significantly higher than the global average [34–36]. In contrast, Hp infection rates in North America has dropped and stomach cancer ASIR was as low as 5.23/100,000 [37]. However, this association is particularly complex in sub-Saharan Africa: despite a high Hp infection rate in the region, the incidence of stomach cancer is not significantly higher due to population youth and competing mortality risks (such as infectious diseases), suggesting that the carcinogenic effect of Hp may be influenced by other factors [38]. 

Diet is another crucial factor, with high-sodium foods and pickled products, common in East Asian diets, being strongly associated with the disease [39, 40]. Smoking also contributes to the risk, with its higher prevalence among men partly explaining their elevated risk of stomach cancer. Excessive alcohol consumption over time further exacerbates the risk [41, 42]. While environmental and lifestyle factors remain the primary causes, genetic predisposition—particularly in cases of familial hereditary stomach cancer—also plays a role [43]. In 2021, smoking accounted for 11.1% of stomach cancer DALYs globally, with this Figure rising to 13.4% in high-middle SDI regions. High-sodium diets contributed to 7.9% of stomach cancer DALYs globally. These findings underscore the importance of controlling smoking and improving dietary habits to prevent stomach cancer.

The variation in the impact of risk factors across SDI regions highlights the necessity for localized interventions. While mortality from stomach cancer has declined in high-SDI regions due to improved screening and treatment, it remains elevated in low-SDI regions, where access to quality care is limited [1]. The global standardized mortality rate fell to 11.24 per 100,000 in 2021; however, this progress has not been uniformed across all regions. Therefore, to reduce the global burden of stomach cancer, priority should be given to expanding access to care, promoting public health policies to reduce smoking, and improving dietary habits. Hp screening, diet adjustment and tobacco and alcohol control are effective preventive measures for high-risk groups, and gastroscopy is an important means to reduce mortality.

The outbreak of COVID-19 at the end of 2019 has had a huge impact on medical care around the world, so here we would also like to discuss the profound impact of COVID-19 on the incidence, diagnosis, treatment and mortality of stomach cancer. The first is delays in endoscopic screening caused by lockdowns and medical disruptions, leading to fewer early stomach cancer diagnoses followed by an increase in cases detected at later stages. With limited treatment options for advanced disease, this shift could lead to increased mortality in the coming years [44]. In addition, surgical delays and interruptions in chemotherapy or targeted therapy affect patient outcomes, while increased hospital burdens lead to delayed or modified treatment regimens [45, 46]. Many patients with stomach cancer, especially those with weakened immune systems, face a higher risk of severe COVID-19 infection, which further affects their prognosis [47]. Although preliminary statistics show a drop in the number of reported stomach cancer cases in 2020, this is largely due to under-diagnosis rather than a real decline in incidence [1]. The long-term impact of the pandemic on stomach cancer mortality remains a major concern, highlighting the need to strengthen screening programmer and improve medical resilience to mitigate future disruptions.

Regional differences in stomach cancer burden are also noteworthy. For example, there is a significant difference in stomach cancer mortality between Southwest Asia and South Asia. With Hp infection rates of 40–50% in Southwest Asia and less than 30% in South Asia, differences in Hp exposure rates directly affect the disease burden [37]. In addition, the traditional diet of Southwest Asia has a higher intake of highly salted foods, which increases the risk of stomach cancer, while the diet of South Asia is mainly vegetarian, where curcumin may have certain anti-inflammatory and antioxidant effects, partially offsetting the effect of Hp infection [48, 49]. In addition, access to care is a key factor: countries in Southwest Asia have a high prevalence of endoscopic screening (about 35%), which improves early diagnosis, while screening rates in parts of South Asia are less than 10%, resulting in more advanced diagnosis [50]. These factors together explain the significant heterogeneity of stomach cancer burden in this region.

Our projections indicate that while the global ASIR for stomach cancer is expected to decline, the entire number of cases will continue to increase. This highlights the critical need for continued efforts in prevention, early discovery, and treatment. While advancements in surgery, chemotherapy, and targeted therapy have improved survival rates to some extent, treatment outcomes remain suboptimal, especially in low- and middle-income countries. Therefore, enhancing the equitable distribution of resources for cancer prevention and control, along with improving diagnostic and therapeutic approaches, is essential to reducing the global burden of stomach cancer. In conclusion, data from the 2021 GBD database indicates that despite declining mortality rates in some developed nations, stomach cancer remains a noteworthy public health challenge, particularly in lower-income regions. By examining incidence, mortality, Disability-Adjusted Life Years (DALYs), and associated risk factors, researchers and policymakers can develop more effective strategies to mitigate the global burden of stomach cancer.

## Strengths and limitations

We conducted a comprehensive study of the global burden of stomach cancer using data from the Global Burden of Disease (GBD) database, encompassing aspects such as incidence, mortality, and Disability-Adjusted Life Years (DALYs), while also examining risk factors, Socio-Demographic Index (SDI) metrics, and projecting future trends. However, several limitations must be acknowledged. First, the quality and availability of data vary significantly across regions and countries. In many underdeveloped and conflict-affected regions, such as sub-Saharan Africa, access to cancer population data is limited due to economic and governmental constraints, which may result in a biased understanding of the stomach cancer burden in these areas. Second, while our study addressed several risk factors for stomach cancer, including smoking, alcohol consumption, and behavioral patterns, these factors were not explored in depth. Additionally, genetic factors that may influence risk were not included in our analysis. Third, our future projections rely on existing trends and patterns, without accounting for variables such as advancements in science and technology, economic growth, and medical progress. Finally, the absence of molecular subtype information in the GBD database limits our understanding of stomach cancer heterogeneity. Aware of these limitations, we emphasize the need to interpret our findings cautiously and call for further research in these areas.

## Conclusion

In summary, this study shows that although the incidence of stomach cancer has declined, significant differences persist between regions with varying levels of socio-economic development. An integrated approach is essential, with priority given to refining access to screening and treatment in low-SDI regions, while promoting early diagnosis and intervention in more affluent areas. Furthermore, prevention and control strategies tailored to the specific socio-economic context of each region, and addressing relevant risk factors, must be implemented to reduce the economic burden on countries. By focusing on reducing modifiable risk factors and improving the clinical and cost-effectiveness of screening programs, we can further mitigate the global burden of stomach cancer.

## Supplementary Information

Supplementary Material 1

## Data Availability

No datasets were generated or analysed during the current study.
