# Supplementary material for: The global burden of stomach cancer and its risk factors from 1990 to 2021: findings from the Global Burden of Disease Study 2021
Source: BMC Public Health. 2025 Aug 6;25:2678. doi: 10.1186/s12889-025-23901-y (PMC12326841; doi:10.1186/s12889-025-23901-y)
Supplement: Supplementary file 1 — Supplementary Material 1 [file 12889_2025_23901_MOESM1_ESM.docx]

Catalogue

Table S1. Estimated annual percentage change in incidence, mortality, and DALYs rates for different regions. 2-4

Table S2：Future predictive value of GBD in stomach cancer incidence and mortality. 5-6

Figure S1：Future Forecasts of GBD of stomach cancer 7

Figure S2: In 1990 and 2021, the number of cases, deaths and DALYs in the world were determined by age and sex. 8

Figure S3: The age-specific burden of stomach cancer, incidence and age-standardized incidence rate 9

Table S1: Estimated annual percentage change in incidence, mortality, and DALYs rates for different regions

| location | ASIR EAPC_CI | ASDR EAPC_CI | DALYs EAPC_CI |
| --- | --- | --- | --- |
| Andean Latin America | -1.41 (-1.56 to -1.26) | -1.59 (-1.74 to -1.44) | -1.73 (-1.88 to -1.59) |
| Australasia | -1.8 (-1.89 to -1.72) | -2.19 (-2.32 to -2.06) | -2.26 (-2.38 to -2.15) |
| Caribbean | -1.28 (-1.35 to -1.21) | -1.41 (-1.48 to -1.33) | -1.25 (-1.35 to -1.16) |
| Central Asia | -2.34 (-2.45 to -2.24) | -2.39 (-2.5 to -2.29) | -2.66 (-2.74 to -2.59) |
| Central Europe | -2.3 (-2.4 to -2.21) | -2.6 (-2.68 to -2.51) | -2.64 (-2.73 to -2.55) |
| Central Europe, Eastern Europe, and Central Asia | -2.7 (-2.76 to -2.64) | -2.94 (-3.01 to -2.87) | -3.16 (-3.25 to -3.08) |
| Central Latin America | -1.96 (-2.04 to -1.88) | -2.17 (-2.25 to -2.09) | -1.99 (-2.07 to -1.9) |
| Central Sub-Saharan Africa | -1.01 (-1.05 to -0.97) | -1.03 (-1.07 to -0.99) | -1.11 (-1.14 to -1.07) |
| China | -1.85 (-2.91 to -0.77) | -3.5 (-4.26 to -2.72) | -2.9 (-3.08 to -2.71) |
| East Asia | -1.63 (-1.8 to -1.47) | -2.52 (-2.73 to -2.31) | -2.87 (-3.05 to -2.68) |
| Eastern Europe | -2.81 (-2.9 to -2.72) | -3.11 (-3.21 to -3.02) | -3.4 (-3.52 to -3.29) |
| Eastern Sub-Saharan Africa | -1.68 (-1.76 to -1.6) | -1.66 (-1.74 to -1.58) | -1.9 (-1.99 to -1.82) |
| Global | -1.81 (-1.87 to -1.75) | -2.26 (-2.34 to -2.18) | -2.54 (-2.62 to -2.46) |
| High SDI | -2.4 (-2.46 to -2.34) | -2.79 (-2.81 to -2.76) | -3.15 (-3.18 to -3.13) |
| High-income | -2.4 (-2.45 to -2.35) | -2.8 (-2.84 to -2.77) | -3.12 (-3.15 to -3.09) |
| High-income Asia Pacific | -3.08 (-3.15 to -3.01) | -3.42 (-3.46 to -3.37) | -3.91 (-3.95 to -3.86) |
| High-income North America | -1.56 (-1.6 to -1.51) | -2.12 (-2.17 to -2.06) | -1.97 (-2.04 to -1.9) |
| High-middle SDI | -1.75 (-1.84 to -1.65) | -2.47 (-2.59 to -2.35) | -2.79 (-2.91 to -2.66) |
| Latin America and Caribbean | -1.85 (-1.92 to -1.78) | -2.03 (-2.09 to -1.96) | -1.96 (-2.03 to -1.89) |
| Low SDI | -1.11 (-1.16 to -1.06) | -1.1 (-1.15 to -1.04) | -1.35 (-1.4 to -1.29) |
| Low-middle SDI | -0.82 (-0.86 to -0.78) | -0.89 (-0.94 to -0.85) | -1.08 (-1.12 to -1.04) |
| Middle SDI | -1.79 (-1.88 to -1.7) | -2.42 (-2.54 to -2.3) | -2.75 (-2.86 to -2.63) |
| North Africa and Middle East | -1.25 (-1.31 to -1.19) | -1.42 (-1.48 to -1.36) | -1.75 (-1.8 to -1.69) |
| Oceania | -0.84 (-0.91 to -0.78) | -0.9 (-0.97 to -0.83) | -0.93 (-1.02 to -0.85) |
| South Asia | -0.9 (-0.96 to -0.85) | -0.94 (-1 to -0.88) | -1.21 (-1.26 to -1.16) |
| Southeast Asia | -1.45 (-1.52 to -1.38) | -1.7 (-1.77 to -1.63) | -1.83 (-1.9 to -1.76) |
| Southeast Asia, East Asia, and Oceania | -1.59 (-1.74 to -1.44) | -2.4 (-2.59 to -2.21) | -2.78 (-2.95 to -2.61) |
| Southern Latin America | -1.43 (-1.55 to -1.32) | -1.67 (-1.78 to -1.56) | -1.71 (-1.82 to -1.6) |
| Southern Sub-Saharan Africa | -0.43 (-0.75 to -0.1) | -0.47 (-0.81 to -0.13) | -0.5 (-0.84 to -0.15) |
| Sub-Saharan Africa | -0.96 (-1.01 to -0.92) | -0.96 (-1.01 to -0.91) | -1.15 (-1.2 to -1.11) |
| Tropical Latin America | -2.16 (-2.22 to -2.11) | -2.31 (-2.37 to -2.26) | -2.28 (-2.33 to -2.22) |
| Western Europe | -2.45 (-2.5 to -2.4) | -3.08 (-3.17 to -2.99) | -3.08 (-3.16 to -3) |
| Western Sub-Saharan Africa | -0.37 (-0.45 to -0.3) | -0.36 (-0.44 to -0.29) | -0.55 (-0.62 to -0.48) |

Table S2：Future predictive value of GBD in stomach cancer incidence and mortality

| Year | DALYs of female | DALYs of female | DALYs of both | Deaths of male | Deaths of female | Deaths of both | Incidence of male | Incidence of male | Incidence of both |
| --- | --- | --- | --- | --- | --- | --- | --- | --- | --- |
| 1990 | 760.43 | 481.6 | 552.85 | 24.01 | 16.17 | 19.05 | 34.32 | 17.97 | 24.65 |
| 1991 | 744.41 | 461.95 | 540.29 | 23.61 | 15.71 | 18.7 | 33.77 | 17.26 | 24.21 |
| 1992 | 731.24 | 445.02 | 529.95 | 23.27 | 15.31 | 18.39 | 33.33 | 16.65 | 23.85 |
| 1993 | 718.4 | 430.11 | 520.29 | 22.93 | 14.96 | 18.11 | 32.91 | 16.05 | 23.52 |
| 1994 | 702.58 | 420.64 | 509.43 | 22.5 | 14.62 | 17.77 | 32.41 | 15.55 | 23.16 |
| 1995 | 687.84 | 408.68 | 497.52 | 22.14 | 14.2 | 17.44 | 31.96 | 15.17 | 22.78 |
| 1996 | 669.43 | 387.97 | 483.55 | 21.65 | 13.74 | 17.01 | 31.35 | 14.6 | 22.29 |
| 1997 | 651.61 | 373.88 | 470.04 | 21.19 | 13.31 | 16.61 | 30.71 | 14.13 | 21.79 |
| 1998 | 639.86 | 362.86 | 460.74 | 20.92 | 13.01 | 16.36 | 30.35 | 13.78 | 21.48 |
| 1999 | 630.44 | 355.15 | 453.91 | 20.71 | 12.79 | 16.17 | 30.03 | 13.4 | 21.23 |
| 2000 | 626.26 | 345.82 | 448.91 | 20.7 | 12.53 | 16.07 | 29.92 | 13.12 | 21.05 |
| 2001 | 616.78 | 335.47 | 440.87 | 20.53 | 12.27 | 15.89 | 29.61 | 12.79 | 20.77 |
| 2002 | 607.97 | 327.2 | 433.8 | 20.35 | 12.04 | 15.71 | 29.35 | 12.53 | 20.54 |
| 2003 | 601.76 | 319.35 | 427.78 | 20.31 | 11.85 | 15.6 | 29.35 | 12.37 | 20.45 |
| 2004 | 594.02 | 310.26 | 420.84 | 20.18 | 11.6 | 15.44 | 29.32 | 12.16 | 20.35 |
| 2005 | 574.93 | 293.11 | 406.56 | 19.65 | 11.17 | 15 | 28.77 | 11.75 | 19.92 |
| 2006 | 544.01 | 274.73 | 384.79 | 18.68 | 10.56 | 14.25 | 27.61 | 11.03 | 19.1 |
| 2007 | 525.4 | 261.38 | 370.86 | 18.14 | 10.13 | 13.8 | 26.98 | 10.72 | 18.61 |
| 2008 | 513.57 | 251.39 | 361.35 | 17.86 | 9.82 | 13.53 | 26.64 | 10.48 | 18.31 |
| 2009 | 502.05 | 241.29 | 352.17 | 17.62 | 9.52 | 13.29 | 26.32 | 10.15 | 18.01 |
| 2010 | 491.49 | 233.02 | 343.82 | 17.38 | 9.24 | 13.06 | 26.01 | 9.86 | 17.73 |
| 2011 | 476.33 | 222.98 | 332.79 | 16.95 | 8.91 | 12.72 | 25.44 | 9.54 | 17.31 |
| 2012 | 463.6 | 214.27 | 323.2 | 16.61 | 8.58 | 12.41 | 24.95 | 9.24 | 16.92 |
| 2013 | 449.51 | 207.3 | 313.35 | 16.25 | 8.32 | 12.13 | 24.41 | 9.01 | 16.53 |
| 2014 | 433.78 | 201.37 | 302.99 | 15.83 | 8.08 | 11.83 | 23.73 | 8.78 | 16.09 |
| 2015 | 423.35 | 195.55 | 296.2 | 15.56 | 7.91 | 11.64 | 23.21 | 8.58 | 15.75 |
| 2016 | 415.23 | 192.2 | 291.06 | 15.4 | 7.78 | 11.53 | 22.8 | 8.49 | 15.5 |
| 2017 | 402.83 | 187.67 | 283.41 | 15.09 | 7.63 | 11.33 | 22.27 | 8.35 | 15.17 |
| 2018 | 394.44 | 184.79 | 278.29 | 14.91 | 7.53 | 11.22 | 21.92 | 8.28 | 14.98 |
| 2019 | 387.11 | 181.56 | 273.43 | 14.79 | 7.42 | 11.13 | 21.63 | 8.2 | 14.79 |
| 2020 | 377.73 | 177.91 | 266.95 | 14.6 | 7.27 | 10.96 | 21.16 | 8.07 | 14.49 |
| 2021 | 370.49 | 169.67 | 262.62 | 14.38 | 7.05 | 10.78 | 20.86 | 9.64 | 14.29 |
| 2022 | 361.6 | 166.1 | 257.17 | 14.19 | 6.9 | 10.63 | 20.46 | 9.5 | 14.05 |
| 2023 | 353.16 | 162.73 | 251.97 | 14.01 | 6.77 | 10.5 | 20.09 | 9.37 | 13.82 |
| 2024 | 344.97 | 159.52 | 246.91 | 13.83 | 6.66 | 10.36 | 19.72 | 9.25 | 13.6 |
| 2025 | 336.98 | 156.43 | 241.95 | 13.63 | 6.53 | 10.21 | 19.36 | 9.13 | 13.37 |
| 2026 | 329.35 | 153.45 | 237.24 | 13.45 | 6.4 | 10.07 | 19.01 | 9.01 | 13.16 |
| 2027 | 322.1 | 150.58 | 232.77 | 13.28 | 6.27 | 9.94 | 18.67 | 8.9 | 12.96 |
| 2028 | 315.06 | 147.8 | 228.44 | 13.11 | 6.16 | 9.82 | 18.35 | 8.78 | 12.76 |
| 2029 | 307.98 | 145.06 | 224.05 | 12.93 | 6.06 | 9.69 | 18.02 | 8.67 | 12.56 |
| 2030 | 300.79 | 142.34 | 219.57 | 12.75 | 5.96 | 9.56 | 17.69 | 8.55 | 12.35 |


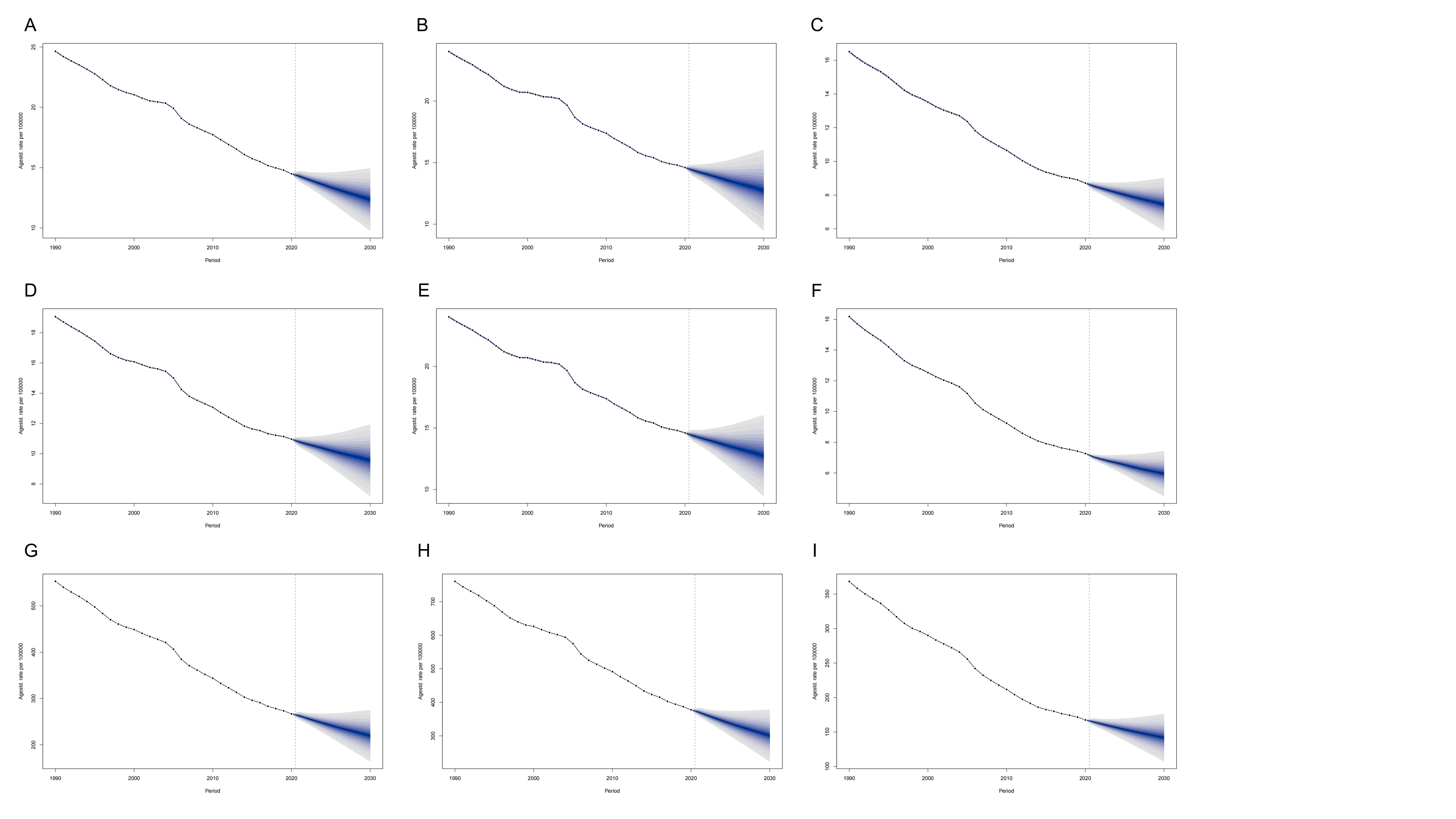


Figure. S1. Future Forecasts of GBD of stomach cancer. (A) ASIR of Both; (B) ASIR of Male; (C) ASIR of Female;(D) ASDR of Both; (E) ASDR of Male; (F) ASDR of Female; (G) Age-Standardized DALYs Rate of Both; (H) Age-Standardized DALYs Rate of Male; (I) Age-Standardized DALYs Rate of Female. DALYs, disability-adjusted life–years. ASIR, Age-standardized incidence rate. ASDR, Age-standardized death rate.


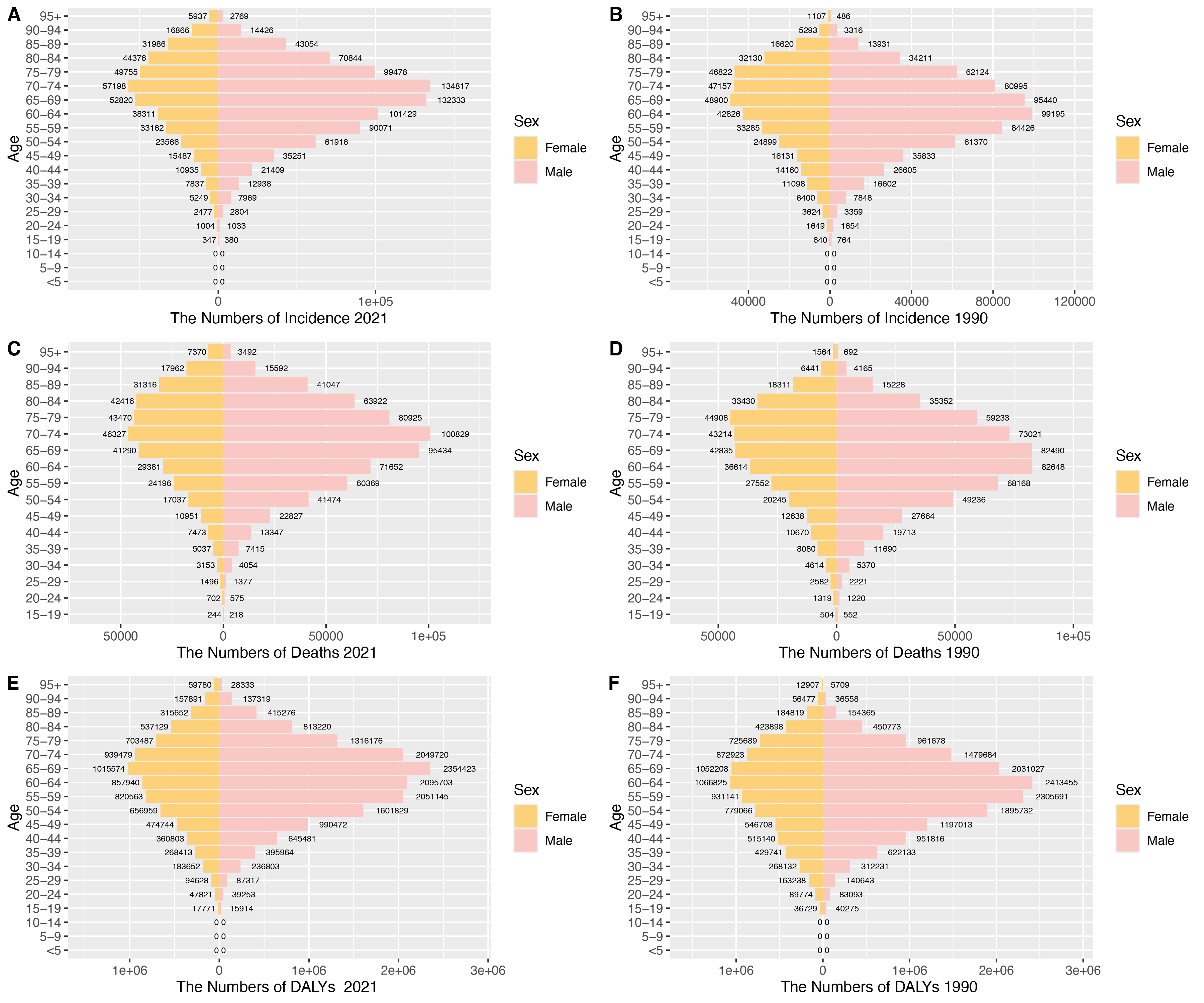


Figure S2：In 1990 and 2021, the number of cases, deaths and DALYs in the world were determined by age and sex.(A)The Number of Incidence 2021;(B) The Number of Incidence 1990;(C) The Number of Deaths 2021;(D) The Number of Deaths 1990;(E) The Number of DALYs 2021;(F) The Number of DALYs 1990.


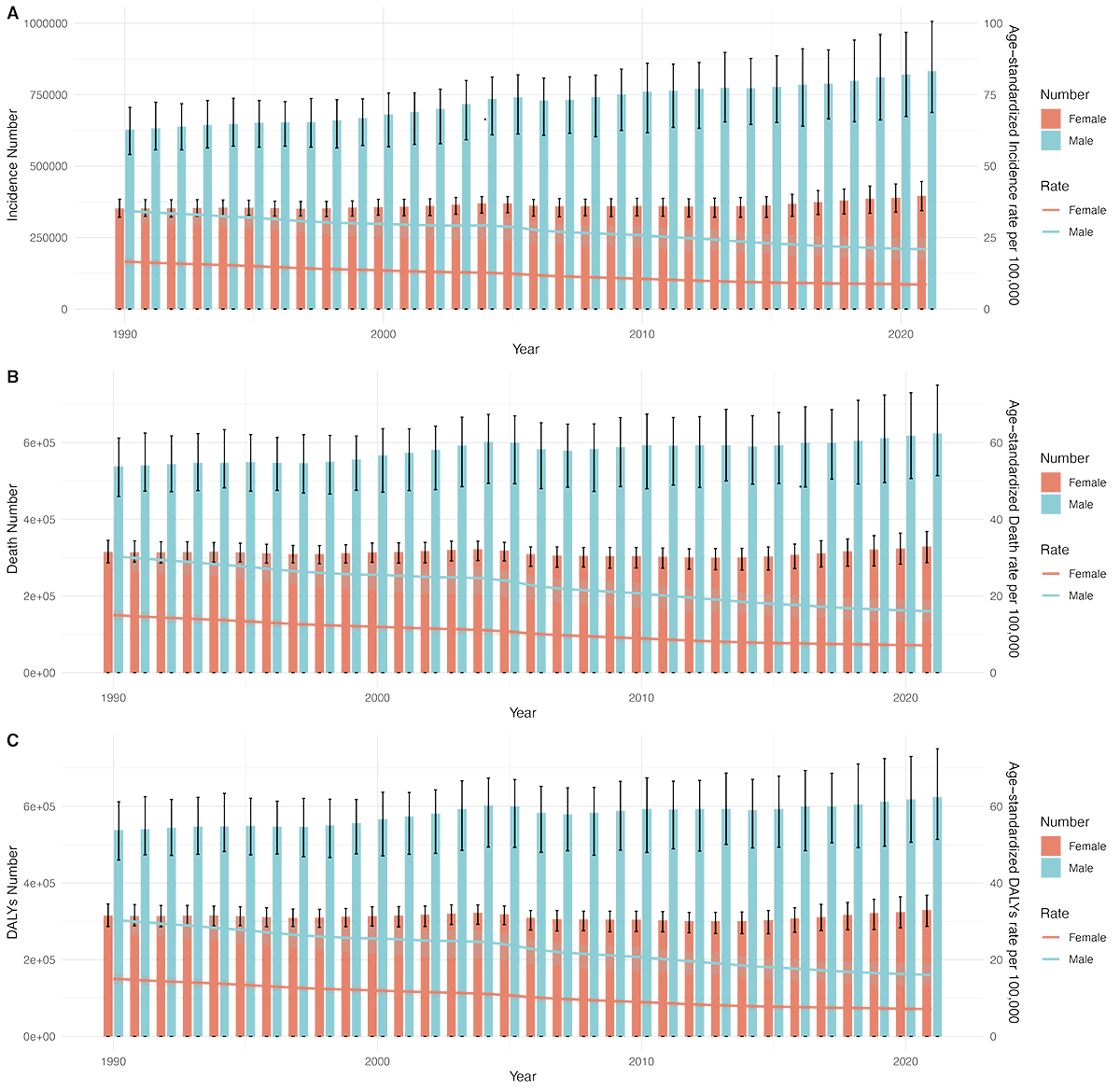


Figure S3**:** The age-specific burden of stomach cancer, incidence and age-standardized incidence rate (A), deaths and age-standardized mortality rate (B), DALYs and age-standardized DALYs rate (C), in 2021. DALYs, disability-adjusted life–years.
